# Exploration of immune checkpoint inhibitors in neoadjuvant therapy of early-stage breast cancer

**DOI:** 10.1097/JS9.0000000000001099

**Published:** 2024-01-19

**Authors:** Tong-Yue Ren, Zhao Bi

**Affiliations:** Shandong Cancer Hospital and Institute, Shandong First Medical University and Shandong Academy of Medical Sciences, Jinan, Shandong, People’s Republic of China

*Dear Editor*,

We recently delved into the enlightening article by Zheng and colleagues in the *International Journal of Surgery*^1^, which evaluated the efficacy and safety of camrelizumab, an IgG4-k PD-1 monoclonal antibody that has shown effectiveness and economy in China, plus chemotherapy as the neoadjuvant therapy (NAT) in early triple-negative breast cancer (TNBC). This article was a single-arm, phase II trial to assess the pathological complete response (pCR) and adverse events (AEs) in patients who received camrelizumab plus chemotherapy as the NAT. The authors showcased that neoadjuvant camrelizumab plus chemotherapy had a high pCR rate (65%) in early-stage TNBC, with an acceptable safety profile. In addition, they found TAP1 and IRF4 may be promising predictive biomarkers for response to the NAT, and aggravated hypoxia and activated glycolysis after the NAT may be associated with the treatment resistance. The innovative strides taken by Zheng *et al*.^1^ and team in this domain are truly commendable and signify that we will apply more immune checkpoint inhibitors (ICIs) into the neoadjuvant setting of TNBC.

Recently, numerous trials have shown that the addition of ICIs to the neoadjuvant setting can increase the efficacy of early-stage TNBC. In the KEYNOTE-522 study, the addition of the anti-programmed cell death protein 1 monoclonal antibody pembrolizumab to neoadjuvant chemotherapy followed by adjuvant pembrolizumab significantly increased pCR and event-free survival (EFS) versus neoadjuvant chemotherapy alone in patients with early-stage TNBC^2^. In the overall study population, the pCR rate was 64.8% (95% CI, 59.9–69.5%) in the pembrolizumab and neoadjuvant chemotherapy group versus 51.2% (95% CI, 44.1–58.3%) in the neoadjuvant chemotherapy alone group. In the subgroup analysis, among the 216 patients enrolled in Asia, there was a bigger difference in pCR with pembrolizumab versus placebo (58.7 vs. 40.0%) and better EFS (91.2 vs. 77.2% at 36 months) compared with the overall study population. And the safety was as anticipated. On the basis of these results, neoadjuvant pembrolizumab plus chemotherapy for the treatment of patients with high-risk, early-stage TNBC has received regulatory approval in Asia.

Considering the difference in pembrolizumab in the Chinese population, we are conducting a phase III clinical trial to evaluate the efficacy and safety outcomes of pembrolizumab plus neoadjuvant chemotherapy versus camrelizumab plus neoadjuvant chemotherapy in the Chinese population with early TNBC. The current result showed that there was no significant difference in pCR between the pembrolizumab group (*n*=42) and camrelizumab group (*n*=45) (63.6 vs. 61.5%, *P*=0.451). Grade 3 to 4 treatment-related AEs occurred in 58.1% of patients receiving pembrolizumab plus neoadjuvant chemotherapy and 56.4% of patients receiving camrelizumab plus chemotherapy. We hope future results could provide evidence for the approval of neoadjuvant camrelizumab plus chemotherapy in the Chinese population with high-risk, early-stage TNBC.

In addition to TNBC, the application of ICIs in the neoadjuvant setting is also being explored among patients with hormone receptor positive/HER2 negative (HR+/HER2−) disease. KEYNOTE-756 is a multicenter, double-blind, randomized clinical trial^3^. The purpose is to assess the efficacy and safety outcome between the addition of pembrolizumab to neoadjuvant chemotherapy versus neoadjuvant chemotherapy alone in patients with early HR+/HER2− disease. The main outcome is pCR at the time of definitive surgery and EFS. The current median follow-up was 33.2 months. The pCR rate was 24.3% (95% CI, 21.0–27.8%) in the pembrolizumab and neoadjuvant chemotherapy group versus 15.6% (95% CI, 12.8–18.6%) in the neoadjuvant chemotherapy alone group. And the breast pCR also achieved a similar result with the main outcome (29.4 vs. 18.2%). The EFS data is not yet mature. These results suggested that HR+/HER2− patients might also benefit from neoadjuvant pembrolizumab therapy. However, the pCR of neoadjuvant pembrolizumab therapy in HR+/HER2− patients is far less than that in TNBC patients. The reason might be that some patients were insensitive to chemotherapy. Neoadjuvant endocrine therapy (NET) has gained increasing attention in recent years. Compared with neoadjuvant chemotherapy, NET had a similar clinical response rate (OR, 1.08; 95% CI, 0.50–2.35; *P*=0.85) and radiological response rate (OR, 1.38; 95% CI, 0.92–2.07; *P*=0.12), but with lower toxicity. The results of the POETIC trial also showed that both a low initial Ki-67 value and a decrease in Ki-67 value caused by NET predict a better clinical response and prognosis^4^. In other words, the Ki-67 index might be an appropriate predictive biomarker. Therefore, we recommend to adopt an ‘adaptive design’ for high-risk, early-stage HR+/HER2− patients^5^. Patients need to receive NET first. Then, those patients who could not benefit from NET can be screened out according to the evaluation of the Ki-67 index. Finally, those patients with insensitive to NET would receive neoadjuvant chemotherapy combined with ICIs to achieve a better therapeutic outcome.

In conclusion, the value of ICIs is gradually being explored in the neoadjuvant setting of early-stage breast cancer. While emphasizing the efficacy of NAT, we should also pay attention to screening out efficacy predictive biomarkers. In clinical practice, we should adopt an ‘adaptive design’, in order to get more benefits.

## Ethical approval

Not applicable.

## Sources of funding

This work was supported by the Shanghai Anti-cancer and Anti-cancer Development Foundation (CYBER-2022- 002) and China Postdoctoral Science Foundation (Grant No. 2022M721987).

## Author contribution

T.-Y.R. and Z.B.: wrote the paper.

## Conflicts of interest disclosure

All authors have stated that they have no conflicts of interest in this work.

## Research registration unique identifying number (UIN)

1. Name of the registry: not applicable.
2. Unique identifying number or registration ID: not applicable.
3. Hyperlink to your specific registration (must be publicly accessible and will be checked): not applicable.

## Guarantor

Zhao Bi.

## Data availability statement

The datasets generated during and/or analyzed during the current study are available from the corresponding author (Zhao Bi) on reasonable request. The corresponding author had full access to all the data in the study and had final responsibility for the decision to submit for publication.
